# Characteristics associated with influenza vaccination uptake in pregnancy: a retrospective cohort study

**DOI:** 10.3399/BJGP.2022.0078

**Published:** 2023-01

**Authors:** Thomas Woodcock, Vesselin Novov, Helen Skirrow, James Butler, Derryn Lovett, Yewande Adeleke, Mitch Blair, Sonia Saxena, Azeem Majeed, Paul Aylin

**Affiliations:** Department of Primary Care and Public Health, Imperial College London, London.; Department of Primary Care and Public Health, Imperial College London, London.; Department of Primary Care and Public Health, Imperial College London, London.; Department of Primary Care and Public Health, Imperial College London, London.; Department of Primary Care and Public Health, Imperial College London, London.; Department of Primary Care and Public Health, Imperial College London, London.; Department of Primary Care and Public Health, Imperial College London, London.; Department of Primary Care and Public Health, Imperial College London, London.; Department of Primary Care and Public Health, Imperial College London, London.; Department of Primary Care and Public Health, Imperial College London, London.

**Keywords:** influenza, pregnancy, primary care, sociodemographic factors, vaccines

## Abstract

**Background:**

Pregnant women are at increased risk from influenza, yet maternal influenza vaccination levels remain suboptimal.

**Aim:**

To estimate associations between sociodemographic and health characteristics and seasonal influenza vaccination uptake among pregnant women, and to understand trends over time to inform interventions to improve vaccine coverage.

**Design and setting:**

Retrospective cohort study using linked electronic health records of women in North West London with a pregnancy overlapping an influenza season between September 2010 and February 2020.

**Method:**

A multivariable mixed-effects logistic regression model was used to identify associations between characteristics of interest and the primary outcome of influenza vaccination.

**Results:**

In total, 451 954 pregnancies, among 260 744 women, were included. In 85 376 (18.9%) pregnancies women were vaccinated against seasonal influenza. Uptake increased from 8.4% in 2010/11 to 26.4% in 2017/18, dropping again to 21.1% in 2019/20. Uptake was lowest among women aged 15–19 years (11.9%; reference category) or ≥40 years (15.2%; odds ratio [OR] 1.17, 95% confidence interval [CI] = 1.10 to 1.24); of Black (14.1%; OR 0.55, 95% CI = 0.53 to 0.57) or unknown ethnicity (9.9%; OR 0.42, 95% CI = 0.39 to 0.46); who lived in more deprived areas (OR least versus most deprived [reference category] 1.16, 95% CI = 1.11 to 1.21); or with no known risk factors for severe influenza.

**Conclusion:**

Seasonal influenza vaccine uptake in pregnant women increased in the decade before the COVID-19 pandemic, but remained suboptimal. Targeted approaches are recommended to reducing inequalities in access to vaccination and should focus on women of Black ethnicity, younger and older women, and women living in deprived areas.

## INTRODUCTION

Seasonal influenza vaccination (SIV) is recommended in pregnant women to reduce the risk of infectious complications and adverse outcomes such as pre-term birth.^1^^–^3 Antenatal SIV is safe and reduces the risk of severe influenza and adverse outcomes for both mother and child.^4^^–^8 However, maternal influenza vaccination levels are suboptimal worldwide.^9^^–^12

In the UK, since 2010, the Joint Committee on Vaccination and Immunisation has recommended that pregnant women get the SIV to provide protection during the winter flu season.^13^ Despite these recommendations, data from Public Health England show that in 2020/21, SIV uptake among pregnant women was only 43.6%.^14^ London remains below the national average with only 36.7% of pregnant women vaccinated in the 2020/21 flu season and only 36.0% in North West London.^15^ Accurate local population estimates are needed to identify which women are less likely to get vaccinated, and inform targeted interventions to increase uptake.

Access to vaccines through strong primary care systems is needed for high maternal uptake.^16^ Misconceptions about the safety and efficacy of antenatal vaccinations play a role in pregnant women being unvaccinated, although recommendation by health professionals improves uptake.^9^^,^^17^^–^20 Several studies have found that younger women (aged <25 years),^21^^,^^22^ women from some minority ethnic groups (for example, Black in UK studies and Black and Hispanic in US studies),^23^^–^28 women living in poorer households,^28^^,^^29^ and those with fewer educational qualifications,^30^ are less likely to have a SIV. However, these are either single-centre studies, limited in their generalisability, or survey-based studies susceptible to sampling bias, recall bias, and response bias. Furthermore, there is a lack of evidence concerning trends in uptake rates since 2015. The COVID- 19 pandemic has also highlighted the importance of maternal vaccination as pregnant women are at increased risk of severe COVID-19 disease and designated a priority group for COVID-19 vaccination in the UK.^31^

To address gaps in the current evidence, this retrospective cohort study used individual-level routinely collected data, covering a diverse regional population, to identify characteristics such as demographics, socioeconomic status, and at-risk conditions associated with uptake; and also, to explore trends in uptake over time independently of these factors.

**Table:** How this fits in

Seasonal influenza vaccination for pregnant women is recommended internationally, yet uptake remains suboptimal. In this study, electronic health record data were used, for a population of 2.3 million patients, to understand which groups of women are less likely to get vaccinated for seasonal influenza during pregnancy. Although uptake increased over the duration of the study, there was significant variation with women less likely to get vaccinated if they were younger than 25, or older than 39 years of age, of Black or unknown ethnicity, living in more deprived areas, or did not have a risk factor for severe disease. Future research should inform tailored programmes to improve vaccine uptake in groups with low uptake, as well as improved access to vaccination services.

## METHOD

### Data source and study cohort

The North West London Discover database is a large de-identified healthcare dataset, containing linked primary, acute, mental health, and community care records for 2.3 million patients registered with a GP in North West London, 1.1 million previously registered, and 208 000 previous residents now deceased.^32^ In this study, data were extracted from primary care records to identify women with ≥1 pregnancy between September 2010 and February 2020. For each pregnancy overlapping an influenza vaccination season the authors further extracted data on maternal influenza vaccination status along with covariates concerning demographics and known risk factors for developing serious complications from influenza infections.

Pregnancies were identified using a method developed for the Clinical Practice Research Datalink (CPRD) primary care database.^33^ Adaptations were necessary because of differences between the Discover and CPRD data, reflecting differences between the underlying GP electronic health record systems. Of 4200 Read Clinical Terms Version (CTV)2 codes used by Minassian *et al*,^33^ 589 are not used in the Discover database. The current study therefore used the remaining 3611 codes to identify all recorded events relating to pregnancy.^33^ This process produced a dataset with one record for each pregnancy, and variables including pregnancy outcome type, and estimated start and end dates.

An influenza season was defined as running from 1 September to the end of February the following year. Pregnancies overlapped with an influenza season if their start date was before or equal to the season’s end date; and their end date was after or equal to the season’s start date.

Pregnancies were included in the analysis if:

- the estimated start date fell between 1 September 2010 and 29 February 2020;
- the woman was aged between 15 and 49 years at the estimated pregnancy start; and
- the pregnancy overlapped with an influenza season.

All included pregnancies were deemed to be in women eligible for SIV.

### Influenza vaccination

For each woman with at least one identified pregnancy, all influenza vaccination records were extracted from the primary care data using the set of Read CTV2 codes specified by Public Health England.^34^ The primary outcome for this study was vaccination status, established for each pregnancy to be either ‘vaccinated’ or ‘not vaccinated’ according to the presence or absence of an influenza vaccination record during the corresponding influenza season, and before the end of the pregnancy. The percentage of vaccinated pregnancies in each season is referred to as the vaccine coverage for that season.

### Covariates

For each pregnancy, the following demographic covariates were extracted: the woman’s age at start of pregnancy, ethnicity, GP practice code (a unique identifier for each GP practice), local authority district name, and Index of Multiple Deprivation (IMD) quintile — a measure of socioeconomic deprivation calculated for small local geographic areas. In addition, data were extracted to establish the presence of the following risk factors at the start of the pregnancy: asthma, chronic respiratory disease, chronic heart disease, chronic kidney disease, asplenia, liver disease, chronic neurological disease, diabetes, immunosuppression, and morbid obesity. These conditions, as well as the Read CTV2 codes used to identify them, were those used in the annual Public Health England assessment of SIV uptake in 2019/20.^34^^,^^35^ In all cases, in this study the authors considered any previous diagnosis of the condition as an indication of the presence of the risk factor.

Technical details of the methods used are presented in Supplementary Appendices S1 and S2, and Supplementary Table S1.

### Analysis

The unit of observation was a pregnancy. For all included pregnancies, distributions of women’s’ characteristics and risk factors were described, by vaccination status. To determine factors associated with SIV uptake, a two-level (pregnancy and GP practice) mixed-effects multivariable logistic regression model was fitted using vaccination status as the outcome. A random intercept term was used, all other terms were fixed effects. The following variables were used as fixed-effect covariates: influenza season, woman’s age (start of pregnancy), ethnicity, IMD quintile, and the presence of at-risk conditions (see above). Regression diagnostics were performed, including a check for collinearity, goodness of fit (marginal and conditional *R*^2^^),^^36^ and examination of residuals. Missing data on ethnicity and IMD were treated as a separate level in the main analysis, and analysed through multiple imputation as a sensitivity analysis (see Supplementary Appendix S2 for further details). Results were reported as odds ratios (ORs) with 95% confidence intervals (CIs), and *P*-values with statistical significance for *P*<0.05. All analyses were conducted using R Statistical Software (version 3.6.1), using the lme4 package (version 1.1-21) to fit mixed-effects models.^37^^,^^38^

## RESULTS

In total, 451 954 pregnancies meeting the inclusion criteria, among 260 744 women, were included in the study. The age of women at the start of pregnancy was most commonly 30–34 years (*n* = 142 965, 31.6%), with women aged 25–39 years accounting for 76.8% of pregnancies (*n* = 347 273) (Table 1). Women were of White ethnicity in 202 823 pregnancies (44.9%), and Asian or Asian British in 135 821 pregnancies (30.1%). The ethnicity of the woman was unknown in 5836 pregnancies (1.3%).

**Table 1.** SIV rates in pregnant women registered with a GP in North West London from September 2010 to February 2020

**Characteristics**	**Pregnancies, *n* (%) (*n* = 451 954)**	**SIV, *n* (%) (*n* = 85 376)**	**Coverage, %**
**Age, years**			
15–19	16 169 (3.6)	1922 (2.3)	11.9
20–24	62 656 (13.9)	10 091 (11.8)	16.1
25–29	123 352 (27.3)	24 096 (28.2)	19.5
30–34	142 965 (31.6)	29 658 (34.7)	20.7
35–39	80 956 (17.9)	15 659 (18.3)	19.3
40–44	22 149 (4.9)	3487 (4.1)	15.7
45–49	3707 (0.8)	463 (0.5)	12.5

**Ethnicity**			
Asian or Asian British	135 821 (30.1)	33 737 (39.5)	24.8
Black or Black British	47 160 (10.4)	6662 (7.8)	14.1
Mixed	14 837 (3.3)	2350 (2.8)	15.8
Other ethnic groups	45 477 (10.1)	8005 (9.4)	17.6
White	202 823 (44.9)	34 047 (39.9)	16.8
Unknown	5836 (1.3)	575 (0.7)	9.9

**IMD quintile**			
1 (most deprived)	65 925 (14.6)	10 107 (11.8)	15.3
2	142 532 (31.5)	26 510 (31.1)	18.6
3	116 715 (25.8)	22 795 (26.7)	19.5
4	66 403 (14.7)	13 534 (15.9)	20.4
5 (least deprived)	32 261 (7.1)	8063 (9.4)	25.0
Unknown	28 118 (6.2)	4367 (5.1)	15.5

**Asthma**			
No	412 283 (91.2)	75 734 (88.7)	18.4
Yes	39 671 (8.8)	9642 (11.3)	24.3

**Chronic respiratory disease**			
No	451 414 (99.9)	85 239 (99.8)	18.9
Yes	540 (0.1)	137 (0.2)	25.4

**Chronic heart disease**			
No	449 455 (99.4)	84 747 (99.3)	18.9
Yes	2499 (0.6)	629 (0.7)	25.2

**Chronic kidney disease**			
No	451 390 (99.9)	85 244(99.8)	18.9
Yes	564 (0.1)	132 (0.2)	23.4

**Liver disease**			
No	450 955 (99.8)	85 145 (99.7)	18.9
Yes	999 (0.2)	231 (0.3)	23.1

**Asplenia**			
No	449 944 (99.6)	84 822 (99.4)	18.9
Yes	2010 (0.4)	554 (0.6)	27.6

**Chronic neurological disease**			
No	450 545 (99.7)	85 037 (99.6)	18.9
Yes	1409 (0.3)	339 (0.4)	24.1

**Diabetes**			
No	448 009 (99.1)	83 772 (98.1)	18.7
Yes	3945 (0.9)	1604 (1.9)	40.7

**Immunosuppression**			
No	450 659 (99.7)	85 015 (99.6)	18.9
Yes	1295 (0.3)	361 (0.4)	27.9

**Morbid obesity**			
No	444 028 (98.2)	83 609 (97.9)	18.8
Yes	7926 (1.8)	1767 (2.1)	22.3

*IMD = Index of Multiple Deprivation. SIV = seasonal influenza vaccination.*

The highest number of pregnancies (*n* = 142 532 pregnancies, 31.5%) were for women living in areas in the second IMD quintile, with fewer pregnancies among women in the most deprived quintile (*n* = 65 925 pregnancies, 14.6%) (Table 1). IMD quintiles 3, 4, and 5 show progressively fewer pregnancies compared to IMD quintile 2, with the fewest number of pregnancies being in the least deprived quintile (*n* = 32 261 pregnancies, 7.1%). IMD was unknown in 28 118 pregnancies (6.2%). The most common risk factor was asthma, affecting women in 39 671 pregnancies (8.8%). The least common risk factors were chronic respiratory disease, chronic kidney disease, and liver disease, each affecting <1000 pregnancies (0.1%–0.2%).

**Figure 1.**
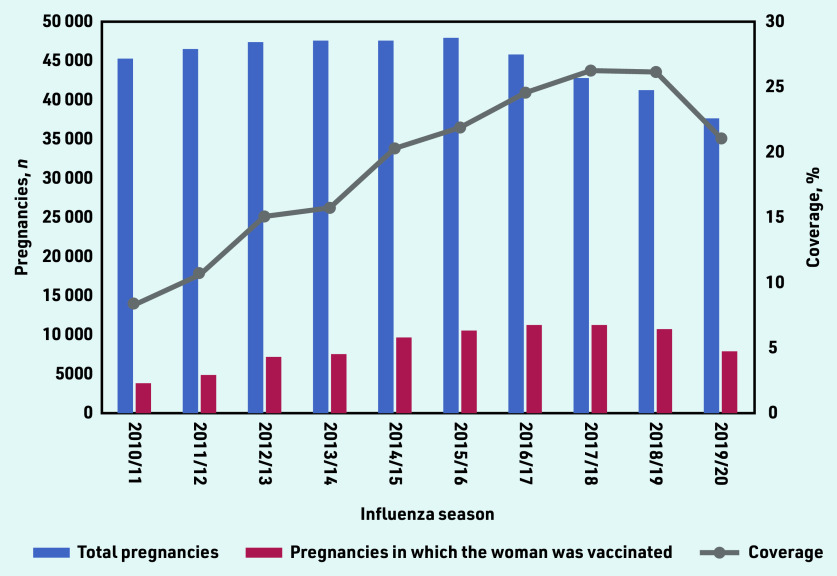
*SIV coverage by influenza season, for women registered with a GP in North West London. The left- hand axis shows counts of the number of pregnancies recorded overlapping with each season, and the number of these in which the woman was vaccinated for seasonal influenza. The right-hand axis shows coverage as a percentage (% pregnancies for which the woman was vaccinated during the overlapping influenza season). SIV = seasonal influenza vaccination.*

For 85 376 of 451 954 pregnancies (18.9%) meeting the inclusion criteria, the woman received SIV during the overlapping influenza season (Table 1). Uptake increased each season from 2010/11 (8.4%, *n* = 3811/45 556 pregnancies) to 2017/18 (26.4%, *n* = 11 322/42 953 pregnancies), and then dropped to 21.1% (*n* = 7976/37 858 pregnancies) in 2019/20 (Figure 1).

Uptake was lowest among women aged 15–19 years (11.9%), rising with age until reaching a peak in those aged 30–34 years (20.7%), before falling again to 15.7% and 12.5% in those aged 40–44 years and 45–49 years, respectively (Table 1). Women of Asian or Asian British ethnicity were most likely to be vaccinated (24.8%) and women of Black or Black British ethnicity (14.1%) and those whose ethnicity was not recorded (‘unknown’, 9.9%) were least likely to be vaccinated. Overall, the proportion of pregnancies where women were vaccinated was highest for those living in less deprived areas (25.0% in the least deprived decile of IMD), and lowest for those living in more deprived areas of North West London (15.3% in the most deprived decile).

Women with each of the influenza risk factors were more likely to be vaccinated than those without the risk factor, although by differing amounts (Table 1). Among pregnancies in women with diabetes, 40.7% (*n* = 1604/3945) were vaccinated, the highest proportion for any risk factor; compared with 18.7% of those without diabetes (*n* = 83 772/448 009). Of all the risk factors, vaccination rates were lowest among pregnancies in women with morbid obesity (22.3%, *n* = 1767/7926), liver disease (23.1%, *n* = 231/999), and chronic kidney disease (23.4%, *n* = 132/564).

In the multivariable logistic regression analysis (Table 2), ORs for age, ethnicity, and IMD quintile showed the same patterns as in the univariate analysis. The effect sizes seen for IMD in this multivariable model are lower than the corresponding univariate effects; for example, the unadjusted ORs for vaccination in IMD quintile 5 versus quintile 1 is 1.89, compared with a direct effect of 1.16 after adjusting in the mixed-effects model. As IMD is likely associated with risk factors included in the model, this suggests that some of the variation in vaccine coverage by IMD may be explained by other covariates. The broad pattern of vaccination by influenza risk factors was also similar, with increased odds of vaccination for those with each risk factor compared with those without. The highest such OR was that for diabetes (2.86, 95% CI = 2.67 to 3.06). The only risk factor for which this effect was not statistically significant was chronic kidney disease.

**Table 2.** Adjusted ORs comparing odds of SIV among pregnant women registered with a GP in North West London from September 2010 to February 2020

**Characteristics**	**OR (95% CI)**	***P*-value**
**Age, years**		
15–19	Reference	
20–24	1.32 (1.25 to 1.40)	<0.001
25–29	1.58 (1.50 to 1.67)	<0.001
30–34	1.68 (1.59 to 1.76)	<0.001
35–39	1.56 (1.48 to 1.64)	<0.001
≥40	1.17 (1.10 to 1.24)	<0.001

**Ethnicity**		
Asian or Asian British	Reference	
Black or Black British	0.55 (0.53 to 0.57)	<0.001
Mixed	0.63 (0.60 to 0.66)	<0.001
White	0.66 (0.65 to 0.68)	<0.001
Other ethnic groups	0.72 (0.70 to 0.74)	<0.001
Unknown	0.42 (0.39 to 0.46)	<0.001

**IMD quintile**		
1 (most deprived)	Reference	
2	1.03 (1.00 to 1.06)	0.06
3	1.06 (1.03 to 1.09)	<0.001
4	1.07 (1.04 to 1.11)	<0.001
5 (least deprived)	1.16 (1.11 to 1.21)	<0.001
Unknown	1.00 (0.96 to 1.04)	0.94

**At-risk group**		
Asthma	1.50 (1.46 to 1.54)	<0.001
Respiratory	1.46 (1.19 to 1.80)	<0.001
Heart	1.43 (1.30 to 1.57)	<0.001
Kidney	1.18 (0.96 to 1.45)	0.13
Liver	1.29 (1.11 to 1.51)	<0.001
Asplenia	1.59 (1.43 to 1.76)	<0.001
Neurological	1.27 (1.12 to 1.45)	<0.001
Diabetes	2.86 (2.67 to 3.06)	<0.001
Immunosuppression	1.84 (1.62 to 2.10)	<0.001
Morbid obesity	1.13 (1.07 to 1.19)	<0.001

**Influenza season, years**	1.14 (1.14 to 1.15)	<0.001

*ORs calculated using logistic regression. Note that as prevalence of at-risk conditions may be influenced by demographics, ORs for demographic variables represent direct effects rather than total effects. OR for influenza season represents the multiplicative increase in odds of SIV for each successive annual influenza season.*

*IMD = Index of Multiple Deprivation. OR = odds ratio. SIV = seasonal influenza vaccination.*

The results of the multiple imputation analysis were consistent with the main analysis and can be found in Supplementary Table S1.

## DISCUSSION

### Summary

This study of over 450 000 pregnancies from 2010 to 2020 found that only one in five pregnancies were in women vaccinated against seasonal influenza, although this rose to one in four towards the end of the study period. Although it is encouraging that Asian or Asian British women and those most at risk from severe influenza were more likely to be vaccinated, wide variation still exists, with only around 1 in 10 pregnant teenagers receiving the seasonal influenza vaccine. Those living in the most deprived areas were half as likely to be vaccinated as those in the least deprived, although some of this effect may be explained by other covariates. Having a chronic condition such as asthma or diabetes, or any of the included risk factors for severe disease, was associated with higher uptake rates. Although this effect was not significant for chronic kidney disease, this may be because of the small number of pregnancies recorded for women with this risk factor.

### Strengths and limitations

The population-level approach used in this study minimised risk of selection bias compared with a sampling approach. The population of North West London is large and demographically diverse, strengthening the external validity of the study. The adapted algorithm for identification of pregnancies provides a rigorous approach, drawing on GP records from all stages of pregnancy, rather than simply counting instances of pregnancy-related codes. The algorithm is widely applicable using data that is available in all GP practices in England. The multivariable regression analysis provides an understanding of the association of each factor with compliance, independently of other covariates; this allows for targeted approaches to improvement.

This study also had some limitations. The data were routinely collected through delivery of primary care, and as such are dependent on the coding practices of primary care teams. The data available did not include some fields used in previous similar studies to identify the outcome of pregnancies, over and above the Read- coded data available in the North West London Discover database. This may have resulted in the outcome of some pregnancies being misattributed; however, pregnancy outcome was not used in this study. Although all available Read-coded vaccination data were included, data were not available to indicate which vaccinations were undertaken within GP practices and which in antenatal clinics. Some vaccinations delivered outside general practices, in community pharmacies for example, may not be recorded. This may mean that vaccination uptake identified through this study is an underestimate; however, the additional data fields were not available across all pregnancies, and this issue is therefore unlikely to bias the findings in relation to the relationships between demographic variables and vaccination uptake.

Completeness of data and appropriate coding of pregnancies will always be a challenge in London with its mobile population, which will have an impact on the accuracy of uptake estimates. Although a range of demographics and risk factors were included in this analysis, access to other variables was not available that might influence uptake of influenza vaccines, for example, political views and influence of social networks. The results, therefore, cannot account for the potential impact of these factors.

### Comparison with existing literature

The level of SIV coverage found in this study was lower than that identified for North West London in a national analysis by Public Health England, although the methods used were different. For example, for the 2019/20 influenza season the current study identified an SIV uptake of 21.1% compared with 34% in the national analysis.^39^ Although in the current study a similar numbers of vaccinations given was found, larger numbers of pregnancies were found in this study compared with the Public Health England analysis, perhaps because of the broader inclusion criteria in this study, which helped minimise bias.^33^ The findings in the current study of lower vaccine uptake in more deprived areas and in women of Black or Black British ethnicity are consistent with previous studies of influenza and other maternal vaccines, including a national study examining social determinants of pertussis and seasonal influenza vaccine uptake.^28^^,^^29^^,^^40^

Walker *et al* reported that women living in the most deprived areas of England had 29% lower odds of being vaccinated against seasonal influenza.^28^ However, their study period was only up until the 2015/16 season. Walker *et al* also reported lower uptake among women of Black and Black British ethnicity and, consistent with this study, that women in clinical risk groups were more likely to be vaccinated. Local studies examining maternal vaccine uptake in a hospital population of pregnant women have reported lower vaccine uptake among Black British and Black ethnicities groups.^41^^,^^42^

### Implications for research and practice

Research is needed to inform tailored programmes for pregnant women living in poorer neighbourhoods and that address variation in vaccine uptake among different ethnicities. Strategies to improve vaccine uptake among pregnant women should not focus exclusively on acceptance of vaccination by pregnant women,^43^ but also improve access to vaccine appointments as recommendations by trusted healthcare providers remain a key driver of maternal vaccine uptake.^44^^,^^45^

The increase in coverage over time in North West London is encouraging, although sustaining this will require more effort as shown by the drop in coverage before the start of the COVID-19 pandemic. Reasons for the increase may include awareness of vaccination in pregnancy over the study period. For example, a study in North West London in 2013/14 found only 63% of women surveyed knew about pertussis vaccine being recommended in pregnancy;^42^ however, in a 2020 national UK survey over 95% of women knew pertussis vaccine was recommended for pregnant women.^46^

From the data available for the current study it is difficult to ascertain how and whether the offer of immunisation was made to all eligible women and the proportion of them that took up the offer. Currently, public health messaging around influenza immunisation for pregnant women is relatively homogeneous. A more nuanced programme, seeking to build relationships between providers and communities over time, and targeted social media messaging is worth considering for younger and older women, and those of Black and Black British ethnicity.^47^^–^49

Women with pre-existing medical conditions are an important group to target because of the additional morbidity and mortality risks associated, and GP information systems are likely to capture this accurately as part of their disease registers, especially if financially incentivised.^50^ There may well be questions to address about who is best placed for discussing immunisation with women, especially as they will often be seen by both primary care and antenatal care teams.

The COVID-19 pandemic had an impact on maternal vaccine programmes in England,^51^ and in the winter 2020/21 seasonal influenza vaccine uptake increased for all groups apart from pregnant women.^15^ Uptake of COVID-19 vaccination among pregnant women is also suboptimal,^52^^,^^53^ and as with seasonal influenza uptake, women from ethnic minorities and women living in more deprived areas are less likely to have been vaccinated against COVID-19.^54^ How the addition of COVID-19 vaccines to the vaccine schedule for pregnant women in the UK influences wider maternal vaccine acceptance and uptake needs monitoring.^55^

In conclusion, these findings provide a robust and detailed understanding of SIV uptake among pregnant women. Uptake of SIV in pregnancy was lower among women living in more deprived areas, women who were younger or older than average, and women of Black or Black British or undocumented ethnicity. There was an increase in uptake over the 10 influenza seasons covered by this study, although there is considerable potential for further increases in uptake, especially with a view to reducing health inequalities.

These findings support targeted interventions to improve uptake of the seasonal influenza vaccine and other vaccines, including COVID-19, offered to pregnant women, along with more accurate recording of vaccination data in medical records.

## Data

The Discover data used in this study are available from Imperial College Partners, but restrictions apply to the availability of these data, which were used under licence for the current study, and so are not publicly available. Researchers wishing to access Discover data can apply as described in Bottle *et al.*^32^
